# The Biochemical Response of Soybean Cultivars Infected by *Diaporthe* Species Complex

**DOI:** 10.3390/plants12162896

**Published:** 2023-08-08

**Authors:** Kristina Petrović, Jovana Šućur Elez, Marina Crnković, Slobodan Krsmanović, Miloš Rajković, Boris Kuzmanović, Đorđe Malenčić

**Affiliations:** 1 Institute of Field and Vegetable Crops, National Institute of the Republic of Serbia, 21000 Novi Sad, Serbia; kristina.petrovic@mrizp.rs (K.P.); slobodan.krsmanovic@agromarket.ba (S.K.); rajkovicmilos@gmail.com (M.R.); 2 Breeding Department, Maize Research Institute, 11185 Belgrade, Serbia; 3 BioSense Institute, University of Novi Sad, 21101 Novi Sad, Serbia; 4 Department of Field and Vegetable Crops, Faculty of Agriculture, University of Novi Sad, 21000 Novi Sad, Serbia; marina.crnkovic@polj.uns.ac.rs (M.C.); kuzmanovic.boris@gmail.com (B.K.); djordje.malencic@polj.uns.ac.rs (Đ.M.); 5 Sector for Plant Nutrition, Agromarket BiH, 76300 Bijeljina, Bosnia and Herzegovina; 6 Department for Research and Development in Agriculture, Institute of Medicinal Plant Research “Dr. Josif Pančić”, 11000 Belgrade, Serbia

**Keywords:** soybean tolerance, *Diaporthe* species, lipid peroxidation (LP), oxidative stress, antioxidant enzymes

## Abstract

Oxidative stress in soybean plants infected with *Diaporthe* isolates was evaluated in order to select (1) the least aggressive inoculation method, (2) to determine the most aggressive *Diaporthe* isolate, and (3) to determine the most tolerant soybean cultivar to this isolate. Based on the present malondialdehyde (MDA) content, the main end product of the lipid peroxidation process, and the biomarker for oxidative stress, the mycelium contact method was chosen as the least aggressive inoculation method, compared to the toothpick method and plug method. The activity of the antioxidant enzymes (superoxide–dismutase (SOD), catalase (CAT), and peroxidase (PX)), the reduced glutathione (GSH) content, and the level of lipid peroxidation (LP) were measured in soybean cv. Sava infected by five different *Diaporthe* species (DPM1F—*D. aspalathi*, DPC/KR19—*D. caulivora*, DPC004NY15—*D. eres*, 18-DIA-SOY-14—*D. gulyae*, and PL157A—*D. longicolla*). The most pathogenic *Diaporthe* species to cv. Sava was *D. eres*. The screening of the antioxidant enzymes activity in the leaves of 12 different soybean cultivars (Altona, Atlas, Capital, Chico, CX134, Favorit, Lakota, McCall, Morsoy, Strain, Rubin, and Victoria) infected with *D. eres* by the mycelium contact inoculation method showed that Capital, McCall, and Morsoy were the cultivars with the highest tolerance to *D. eres*, followed by Chico, Favorit, Lakota, and Rubin. The most sensitive cultivars were Atlas, CX134, Victoria, and Strain.

## 1. Introduction

Soybean (*Glycine max* (L.) Merrill) is one of the most important oil crops globally and has tremendous importance as a food-grade legume. Having a 53% global production share of all oilseed crops, soybean is highly significant in most agricultural production systems in major countries, including the USA, China, Brazil, Argentina, and India [1]. In 2021, Serbia was the 15th largest producer of soybeans, with 719,370 metric tons, expected to reach 824,690 metric tons by 2026 (https://www.reportlinker.com/clp/country/3685/726451 (accessed on 1 July 2023)). Soybean is used for livestock feeding and edible and industrial oil and is a high-protein food crop for human consumption.

**Figure 1:**
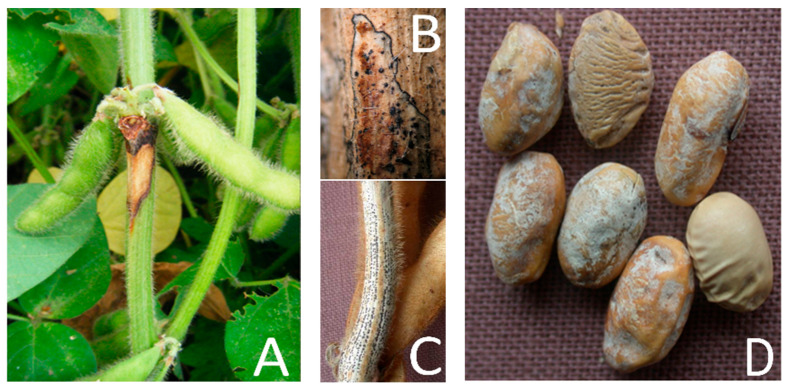
Characteristic symptoms of *Diaporthe* species on soybean: (**A**) stem canker caused by *D. caulivora*; (**B**) black zone lines on the lower portion of a soybean stem caused by *D. longicolla*; (**C**) pod and stem blight caused by *D. sojae* and *D. longicolla,* and (**D**) *Diaporthe* seed decay caused by complex species.

More than 200 phytopathogenic microorganisms (primarily fungi) have been detected on soybean [2], while approximately 30 species can cause significant economic damage to soybean production [3]. However, many phytopathogenic fungi attacking plants during the growing season or existing on the soybean seed can reduce the yield, the nutritional content, the germination, and the seed vigor. Species from the *Diaporthe* genus are one of the most harmful fungal pathogens that can limit soybean production and cause varying types of diseases (e.g., soybean stem canker, pod and stem blight, and seed decay) [4] (Figure 1).

It has been reported that several different species of *Diaporthe* cause complex diseases on soybean stem and seed [5,6]. *Diaporthe longicolla* is the primary agent of seed decay (Figure 1D). It causes pod and stem blight as well, together with *D. sojae* [7,8] (Figure 1C), and black zone lines symptom on stems [9,10] (Figure 1B). However, soybean stem canker is the most harmful stem disease (Figure 1A), especially when the canker lesions develop early, resulting in plant wilting and death [11]. In addition, warm and wet weather conditions, especially during pod filling and maturation, are suitable for infection by pathogen and disease development [12]. Soybean stem canker is mainly caused by two different species, *D. caulivora* (northern stem canker) and *D. aspalathi* (southern stem canker), which seem to be geographically limited to some degree, but their ranges may overlap [13,14,15,16]. Additionally, it was reported recently that *D. gulyae* is also a causal agent of stem disease [17].

In Serbia, the most economically important soybean stem disease is the northern stem canker, caused by *D. caulivora* [18]. The disease intensity varies depending on the year, locality, and cultivar. Out of the total soybean production in Serbia, 87% is grown in Vojvodina (https://ipad.fas.usda.gov (accessed on 1 July 2023)), where moderate infection by *D. caulivora* can reduce the yield by 9–20%. However, over 50% of prematurely wilted plants have been recorded in certain years. Occasionally, epiphytotic disease occurrences were noted, especially in rainy years, where 70–80% of diseased plants occurred (Petrović, personal observations), which affected the yield drastically. The yield of prematurely wilted plants is 50–62% (depending on the cultivar) lower than disease-free ones. However, summer seasons with high temperatures are becoming more frequent in Serbia and the region. These conditions are favorable for developing southern stem canker disease. Therefore, *D. aspalathi,* as well as other *Diaporthe* species, can be expected on soybean in this part of Europe.

Strategies for soybean stem canker management, such as biological control and fungicide application, have shown limited success (https://cropwatch.unl.edu/plantdisease/soybean/stem-canker (accessed on 1 July 2023)). Because of that, growing soybean genotypes tolerant to stem canker is the most sustainable strategy for protecting high yields and seed quality. Moderate levels of resistance to southern stem canker have been reported from the field and greenhouse inoculations [19]. However, to date, little has been done to resolve the genetic resistance to northern stem canker.

In response to the attack of *Diaporthe* pathogens, the first activated mechanism within soybean plants is the synthesis of pathogenesis-related proteins, often called PR proteins. Members of the PR protein family have enzymatic activities, including β-1,3-glucanase (PR-2), chitinase (PR-3, -4, -8, and -11), endoproteinase (PR-7), peroxidase (PR-9), or ribonucleases (PR-10), and have been shown to exhibit either antibacterial or antifungal activity [4,20,21]. The accumulation of these proteins at the site of pathogen infection helps plants defend themselves against the attacks from these pathogens. Moreover, the accumulation of phytoalexin glyceollin, mediated by the synthesis of nitric oxide, represents the response of soybean plants to the attack of pathogens of the *Diaporthe* complex [22].

During the preliminary study of this research, three different inoculation methods (mycelium contact, toothpick, and plug), previously reported as effective techniques to assess the aggressiveness of *Diaporthe* isolates on soybean [23]) were tested using the three most pathogenic *Diaporthe* species (*D. aspalathi*, *D. caulivora*, and *D. longicolla*) and cv. Sava, in order to select the least aggressive method for further examination. The first goal was to investigate the sensitivity of soybean plants (cv. Sava) to five *Diaporthe* isolates identified as *D. aspalathi* (DPM1F), *D. caulivora* (DPC/KR19), *D. eres* (DPC004NY15), *D. gulyae* (18-DIA-SOY-14), and *D. longicolla* (PL157A). The most pathogenic isolate was chosen for further research. The second goal was to compare the sensitivity of 12 soybean cultivars from different maturity groups (MG) (Favorit MG 000, Altona MG 00, Capital MG 00, Chico MG 00, McCall MG 00, Morsoy MG 00, Strain MG 00, Atlas MG 0, CX134 MG I, Lakota MG I, Victoria MG I, and Rubin MG II) to the most pathogenic isolate in order to select soybean cultivars with the highest tolerance. The biochemical parameters of the oxidative stress were measured: the activity of antioxidant enzymes (superoxide–dismutase (SOD), catalase (CAT), and peroxidase (PX)), the reduced glutathione (GSH) quantity, and lipid peroxidation (LP).

## 2. Results

### 2.1. Preliminary Study

Table 1 presents the malondialdehyde (MDA) content, the main end product of the LP process and the biomarker for oxidative stress, in soybean plants infected with three *Diaporthe* isolates separately, by three different inoculation methods (mycelium contact, toothpick, and plug). The MDA accumulation was higher in soybean plants infected with *Diaporthe* isolates using the toothpick and plug methods, compared to mycelium contact. The difference in the MDA content between the examined methods was particularly noticeable in soybean plants infected with *D. aspalathi* and *D. longicolla*. The difference in the MDA content between the examined methods was also observed in the control plants. The least aggressive method, the mycelium contact method, was chosen for the research.

### 2.2. Research 1. Sensitivity of Soybean Plants (cv. Sava) to Diaporthe Isolates

**Figure 2:**
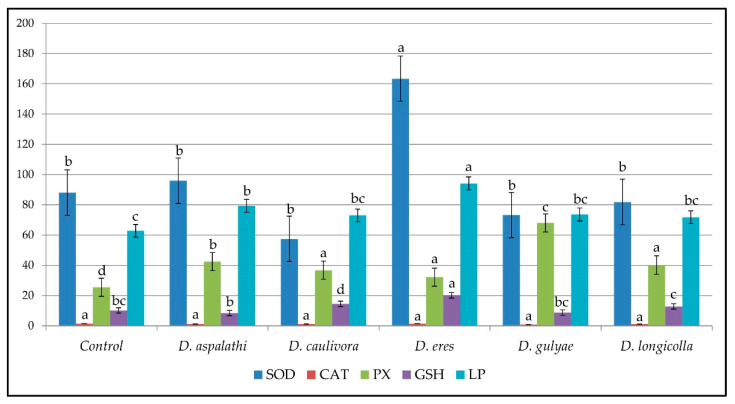
The activity of the antioxidant enzymes SOD, CAT, and PX (U/g FW), the GSH quantity (µmol GSH/g FW), and the MDA content (nmol MDA/g FW) in the leaves of soybean plants (cv. Sava) infected with *Diaporthe* isolates by the mycelium contact inoculation method. a–d Values without the same superscripts within each column differ significantly (*p* < 0.05).

The results of the antioxidant enzyme activity (SOD, CAT, and PX), GSH quantity, and MDA content in the leaves of soybean plants (cv. Sava) infected with five *Diaporthe* species separately, by the mycelium contact inoculation method are shown in Table 2 (raw data available at https://doi.org/10.5281/zenodo.5669484 (accessed on 1 July 2023)), followed by visual presentation of the data (Figure 2). According to Duncan’s multiple range tests, a significant increase in the activity of SOD was detected in the soybean leaves infected with *D. eres* (163.39 ± 12.69 U/g FW), compared to the control plants (88.10 ± 16.26 U/g FW). No significant difference was detected in the activity of CAT, while a significant increase in PX activity was detected in all treatments. The significant induction of GSH production was detected in the leaves of soybean infected with *D. eres* (20.20 ± 0.16 µmol GSH/g FW), compared to the GSH content in the control plants’ leaves (10.15 ± 0.15 µmol GSH/g FW). The MDA content was higher in all treatments compared to the control, but the highest amount of MDA was recorded in the soybean leaves infected with *D. eres* (94.21 ± 11.19 nmol MDA/g FW), compared to the MDA content in the control plants (62.75 ± 0.52 nmol MDA/g FW).

### 2.3. Research 2. Sensitivity of Different Soybean Cultivars to D. eres

**Figure 3:**
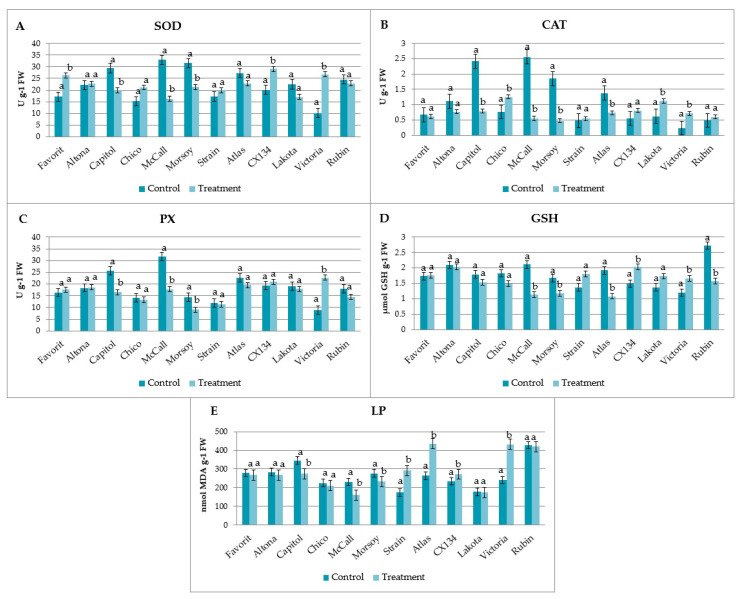
The activity of the antioxidant enzymes (U/g FW): SOD (**A**), CAT (**B**), and PX (**C**), the GSH quantity (µmol GSH/g FW) (**D**), and the MDA content (nmol MDA/g FW) (**E**) in the leaves of different soybean cultivars infected with *D. eres* by the mycelium contact inoculation method. ^a,b^ Values without the same superscripts within each column differ significantly (*p* < 0.05).

The results of the antioxidant enzymes activity (SOD, CAT, and PX), the GSH quantity, and the MDA content in the leaves of 12 soybean genotypes from different maturity groups infected with *D. eres* by the mycelium contact inoculation method are shown in Table 3, followed by a visual presentation of the data (Figure 3).

Soybean cv. Altona did not show statistically significant differences in the investigated oxidative stress parameters compared to its control. In cvs. Chico, Favorit, Lakota, and Rubin, statistically significant differences were detected in one of five investigated oxidative stress parameters. A statistically significant increase in SOD activity was detected in cv. Favorit (26.31 ± 0.74 U/g FW), higher CAT activity was detected in cvs. Chico and Lakota (1.25 ± 0.22 U/g FW and 1.12 ± 0.06 U/g FW, respectively), while lower GSH content was measured in cv. Rubin (1.57 ± 0.16 µmol GSH/g FW), compared to their controls. No increase in lipid peroxidation was observed in any of these cultivars.

Among all the investigated soybean cultivars, in cvs. Atlas, CX134, Victoria, and Strain, a statistically significantly higher MDA content was measured compared to their controls (435.11 ± 8.72 nmol MDA/g FW, 272.37 ± 2.23 nmol MDA/g FW, 431.82 ± 1.22 nmol MDA/g FW, and 291.67 ± 5.10 nmol MDA/g FW, respectively). Furthermore, in cv. Victoria, a statistically significant increase in the SOD, CAT, and PX activity was detected, while a statistically significant increase in the SOD and a decrease in the CAT activities were detected in cv. CX134 and cv. Atlas, respectively.

A lower level of lipid peroxidation process was measured in soybean cvs. Capitol, McCall, and Morsoy followed by the decrease in the activity of the antioxidant enzymes SOD, CAT, and PX and a lower GSH content compared to their controls.

## 3. Discussion

This study initially analyzed three different inoculation methods (mycelium contact, toothpick, and plug), previously reported as effective techniques in testing the aggressiveness of different *Diaporthe* isolates on soybean [23], aiming to select the least aggressive method for further examination. Based on the MDA content, a substance produced by membrane lipids in response to reactive oxygen species (ROS), in the leaves of soybean plants (cv. Sava) infected with the three most pathogenic *Diaporthe* species (*D. aspalathi*, *D. caulivora*, and *D. longicolla*), the mycelium contact method was chosen as the least aggressive method.

Afterward, this inoculation method was used for testing the sensitivity of cv. Sava (previously reported as a suitable host for the pathogenicity testing of *Diaporthe* species [24]) to five different *Diaporthe* species recovered from soybean stem (*D. aspalathi*, *D. caulivora*, *D. eres*, *D. gulyae,* and *D. longicolla*). The soybean cv. Sava showed the highest sensitivity to *D. eres* with the highest amount of MDA, the highest production of GSH, the highest activity of SOD, and a high activity of PX, compared to the other tested *Diaporthe* species. The SOD activity plays a central role in the defense against oxidative stress, and PXs are widely accepted as enzymes of stress and can act as effective quenchers of reactive intermediary forms of O_2_ and peroxy radicals under stressed conditions. On the other hand, no significant difference was detected in the CAT activity. The peroxisomes are major sites of H_2_O_2_ production, and CAT scavenges H_2_O_2_ generated in this organelle. CATs have a very fast turnover rate, but stresses that reduce the rate of protein turnover also reduce the CAT activity. Environmental stresses cause either an enhancement or depletion of antioxidant enzyme activity, depending on the intensity, duration, and type of stress [25]. It seems that soybean cv. Sava was unable to counteract *D. eres*. Thus, *D. eres* was chosen for further research.

The results of the antioxidant enzymes’ activity in the leaves of 12 soybean cultivars from different maturity groups (Favorit MG 000, Altona MG 00, Capital MG 00, Chico MG 00, McCall MG 00, Morsoy MG 00, Strain MG 00, Atlas MG 0, CX134 MG I, Lakota MG I, Victoria MG I, and Rubin MG II) infected with *D. eres* by mycelium contact inoculation method are in line with the study by Fortunanto et al. [26], who reported an increase in antioxidant enzymes’ activity in soybean plants infected by necrotrophic fungus *Corynespora cassiicola*, e.g., SOD–one of the most important antioxidant enzymes representing the first line of defense against ROS, contributed to the lower concentration of ROS and the reduced damage to the plant cell plasma membrane as indicated by the lower concentration of MDA. On the other hand, Kuzniak and Skłodowska [27] reported that *Botrytis cinerea* triggered significant changes in the tomato peroxisomal antioxidant system leading to a collapse of the protective mechanism at the advanced stage of infection. Mandal et al. [28] reported that an increase in lipid peroxidation in tomato plants infected by *Fusarium oxysporum* was detected.

The activity of antioxidant enzymes is frequently used as an indicator of oxidative stress in plants. Under oxidative stress, the oxidative damage of cell membranes is observed. The main biomarker for oxidative stress is the MDA content, the main end product of the lipid peroxidation process. Thus, the activity of the antioxidant enzymes and the MDA content were investigated to choose the most pathogenic *Diaporthe* isolate and select the soybean cultivars with higher tolerance to the most pathogenic *Diaporthe* isolate. The soybean cv. Sava showed different sensitivity to the tested *Diaporthe* isolates with the highest sensitivity to *D. eres*. Furthermore, *D. eres* had different effects on the tested soybean cultivars. A significantly higher accumulation of MDA in the leaves of soybean cvs. Atlas, CX134, Victoria, and Strain infected with *D. eres* indicated that *D. eres* caused oxidative damage of the membranes in these cultivars. Based on these results, cvs. Atlas, CX134, Victoria, and Strain are marked as cultivars with higher sensitivity to the tested *Diaporthe* isolate, among all the investigated soybean cultivars. In the cv. Altona, followed by cvs. Chico, Favorit, Lakota, and Rubin there was no significant difference in the MDA content in the treatment compared to the control and the cvs. Altona, Chico, Favorit, Lakota, and Rubin are marked as tolerant. The cultivars McCall, Morsoy, and Capitol are marked as highly tolerant.

It was reported that early-maturing cultivars (MG ’000-0’) were tolerant to *Diaporthe* species in the field and usually responded with mild symptoms (stem blight), while the late-maturing cultivars (MG I-II) were susceptible, and the symptoms were manifested as premature wilting of plants [11]. Based on this, it was concluded that early-maturing cultivars avoid *Diaporthe* infection. However, the results of this research have shown that early-maturing cultivars such as cvs. Favorit (MG 000), Altona (MG 00), Capitol (MG 00), Chico (MG 00), McCall (MG 00), and Morsoy (MG 00) are tolerant, but others are very susceptible, such as cvs. Strain (MG 00) and Atlas (MG 0). Similarly, late-maturing cultivars can be tolerant, such as Lakota (MG I) and Rubin (MG II), while CX134 (MG I) and Victoria (MG I) are susceptible cultivars. Recently, it was reported that soybean cvs. Atlas (MG 0) and Rubin (MG II) are tolerant to charcoal rot disease caused by *Macrophomina phaseolina* (Tassi) Goid, while Favorit (MG 000) and Victoria (MG I) are marked as susceptible cultivars [29]. In the current study cvs. Favorit (MG 000) and Rubin (MG II) showed tolerance to *D. eres*, while cvs. Atlas (MG 0) and Victoria (MG I) were susceptible. This indicates that late-maturing cv. Rubin (MG II) is favorable for sustainable soybean production in the agroecological environment in southeastern Europe.

The research on sunflower (*Helianthus annuus* L.) demonstrated that isolates of *D. helianthi* and *D. gulyae* were similar in their aggressiveness. Out of 49 accessions, 13 were less susceptible to *D. helianthi*, while four were less susceptible to *D. gulyae*, compared to HA 288. Only one accession (PI 552939) was observed to be significantly less vulnerable to both *D. helianthi* and *D. gulyae* when compared with HA 288 [30].

Mena et al. [4] investigated the sensitivity of the SSC-susceptible soybean cv. Williams (PI 548631) to *Diaporthe* isolates taken from different plants from various regions of Uruguay. The results indicated that the stems were more sensitive than the leaves, and the development of symptoms in the leaves was slower than in stems. The first stem canker symptoms were observed at three days post-inoculation, while canker lesions progressed in the stems leading to leaf withering at seven days post-inoculation.

## 4. Materials and Methods

### 4.1. Preliminary Study

Preliminary research involved selecting the least aggressive inoculation method. *Diaporthe aspalathi*, *D. caulivora*, and *D. longicolla*, recovered from soybean stem, were selected for this trial, because they were recognized as the most virulent species in previous research [4]. Also, three standard inoculation methods: mycelium contact, toothpick, and plug were selected, based on their most common use in the tolerance study of soybean cultivars to diseases caused by the *Diaporthe* complex [23]. To obtain inoculum, three *Diaporthe* isolates identified as *D. aspalathi* (DPM1F recovered from soybean stem in Georgia, USA), *D. caulivora* (DPC/KR19 recovered from soybean stem in Rimski Šančevi, Serbia), and *D. longicolla* (PL157A recovered from soybean stem in Rimski Šančevi, Serbia) were grown on PDA at 22 °C for 10 days under 12 h of alternating light and dark conditions.

**Figure 4:**
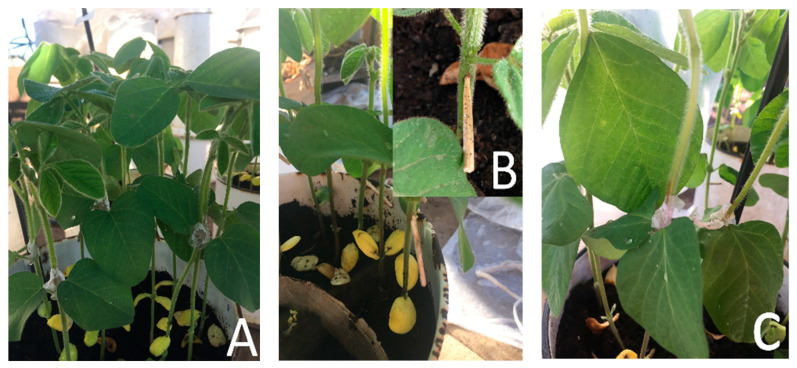
Inoculation methods applied in the preliminary study: (**A**) mycelium contact method that was used for testing the aggressiveness of different *Diaporthe* species (*Research 1*) and screening soybean tolerance to *D. eres* (*Research 2*), (**B**) toothpick method, and (**C**) plug method.

For all the inoculation methods, five soybean plants (cv. Sava that was reported in the previous research as a suitable host for pathogenicity testing of *Diaporthe* species [24]) were inoculated at the V2 growth stage. For the mycelium contact method (Figure 4A), a mycelial plug (~5 mm diameter) was taken from the margin of a 10-day-old *Diaporthe* culture and placed in contact with the stem portion of the soybean plants ~1–2 cm above the cotyledons. Parafilm was applied over the top of the plug to avoid dehydration. For the toothpick method (Figure 4B), autoclaved toothpicks were placed on PDA plates containing the *Diaporthe* culture, and the plates were incubated at 22 °C for 15 days under 12 h of alternating light and dark conditions. After 15 days, when the toothpicks were colonized by the fungus, they were inserted into the stems of the soybean plants ~1–2 cm above the cotyledons. The inoculation site was sealed with petroleum jelly. For the plug method (Figure 4C), a cut was made ~1–2 cm above the cotyledons on the stem with a lancet tool. A mycelial plug (~5 mm diameter) was taken from the margin of a 10-day-old *Diaporthe* culture and placed into the cut. The cut was covered with parafilm. After three days, samples were taken. To determine the lipid peroxidation intensity, 2 g of fresh leaves were homogenized in 10 mL of phosphate buffer (0.1 M, pH 7.0) prepared in-house and centrifuged. The MDA content in the supernatants was determined using the thiobarbituric acid (TBA) test. The absorbance was recorded at 532 nm using a UV/VIS spectrophotometer (Thermo Scientific Evolution 220, Waltham, MA, USA). The least aggressive inoculation method was chosen based on the results for the accumulation of MDA. The reason for this is the fact that in nature, infectious spores often penetrate the plant through leaves or mechanical injuries on the soybean stem (a spot where leaf petiole is broken) [31]. However, the production of spores in most *Diaporthe* species is complicated and sometimes impossible because *Diaporthe* species very fast become sterile in agar plates and lose the ability to form spores. For this reason, it is much easier to use mycelia as inoculum for testing pathogenicity or resistance. Moreover, it is preferable to carry out less aggressive inoculation methods in order to imitate natural infections [32].

### 4.2. Research 1

Using the mycelium contact method described by Ghimire et al. [23], the aggressiveness of the five isolates identified as *D. aspalathi* (DPM1F recovered from soybean stem in Georgia, USA), *D. caulivora* (DPC/KR19 recovered from soybean stem in Rimski Šančevi, Serbia), *D. eres* (DPC004NY15 recovered from soybean stem in St. Lawrence County, NY, USA), *D. gulyae* (18-DIA-SOY-14 recovered from soybean stem in Brookings, SD, USA), and *D. longicolla* (PL157A recovered from soybean stem in Rimski Šančevi, Serbia) was determined by the inoculation of five soybean plants of cv. Sava at the V2 growth stage. After three days, samples were taken, and the activity of the antioxidant enzymes (SOD, CAT, and PX), the GSH quantity, and the MDA content in the leaves was determined. Based on the results, the most aggressive *Diaporthe* isolate (*D. eres*) was chosen for further research.

### 4.3. Research 2

Using isolate DPC004NY15, identified as *D. eres*, 10 plants of each of 12 soybean cultivars (Altona, Atlas, Capital, Chico, CX134, Favorit, Lakota, McCall, Morsoy, Strain, Rubin, and Victoria) from the collection of the Institute of Field and Vegetable Crops, Novi Sad, Serbia were inoculated at the V2 growth stage by the mycelium contact method described by Ghimire et al. [23]. After three days, samples were taken, and the activity of the antioxidant enzymes (SOD, CAT, and PX), the GSH quantity, and the MDA content in the leaves were determined. Based on that, the most tolerant soybean cultivars to *D. eres* were selected.

To determine the biochemical parameters, 1 g of fresh leaves was taken from 12 inoculated soybean cultivars and the control and homogenized in 10 mL of the phosphate buffer (0.1 M, pH 7.0). These extracts were centrifuged, and the supernatants were obtained. The biochemical analyses were performed spectrophotometrically using a UV/VIS spectrophotometer (Thermo Scientific Evolution 220, Waltham, MA, USA).

The SOD (EC 1.15.1.1) activity was estimated according to Mandal et al. [28] with the modification based on the inhibition of the photochemical reduction of the nitro blue tetrazolium (NBT) chloride. One unit of the SOD activity was taken as that amount of enzyme required to inhibit the reduction of the NBT by 50%. The enzyme activity was expressed as unit per g of fresh weight (U/g FW). The CAT (EC 1.11.1.6) activity was assayed by measuring the disappearance of the H_2_O_2_ according to Sathya and Bjorn [33]. The decrease in absorbance at 240 nm was observed. The enzyme extract was added to the assay mixture containing 50 mM of potassium phosphate buffer (pH 7.0) prepared in-house and 10 mM of H_2_O_2_. The enzyme activity was expressed as U per gram of fresh weight (U/g FW). The PX (EC 1.11.1.7) activity was measured using pyrogallol as substrate, according to Morkunas and Gmerek [34]. This method is based on the purpurogallin content measurement (a product of pyrogallol oxidation). The enzyme extract was added to the assay mixture containing 180 mM pyrogallol and 2 mM H_2_O_2_. The absorbance was recorded at 430 nm. The enzyme activity was expressed as U per gram of fresh weight (U/g FW). The GSH quantity was determined according to Sedlak and Lindsay [35] and expressed as μmol GSH per gram of fresh weight (µmol GSH/g FW). The content of MDA, the end product of the lipid peroxidation process, was determined by measuring the absorbance at 532 nm using the TBA test [28]. The total amount of TBA-reactive substances was given in nmol of MDA equivalents per g of fresh weight (nmol MDA/g FW).

All measurements were performed in triplicate. The values of the biochemical parameters were expressed as the mean ± standard error of mean and tested by ANOVA, followed by a comparison of the means by Duncan’s multiple range test (*p* < 0.05). Data were analyzed using Statistica software [36].

## 5. Conclusions

According to the obtained results, it can be concluded that biochemical markers are useful for the fast testing of inoculation method aggressiveness. The accumulation of MDA showed that the mycelium contact method was less aggressive than the toothpick and plug methods. It is important to choose the inoculation method that most imitates natural infection. Otherwise the results may show a false picture regarding plant reaction to pathogens. Additionally, using the set of biochemical parameters (the activity of antioxidant enzymes: SOD, CAT, and PX, then GSH quantity and LP presented as MDA content) of soybean cv. Sava infected with five different *Diaporthe* species recovered from soybean stem (DPM1F—*D. aspalathi*, DPC/KR19—*D. caulivora*, DPC004NY15—*D. eres*, 18-DIA-SOY-14—*D. gulyae*, and PL157A—*D. longicolla*), it was concluded that *D. eres* was the most pathogenic species. Afterward, the set of oxidative stress responses of 12 screened soybean cultivars with different maturity groups to *D. eres* tolerance showed the most sensitive cultivars were Strain (MG 00), Atlas (MG 0), CX134 (MG I), and Victoria (MG I). The cultivar with a higher tolerance was Altona (MG 00), followed by cvs. Chico (MG 00), Favorit (MG 000), Lakota (MG I), and Rubin (MG II). The soybean cultivars with the highest tolerance to *D. eres* were McCall (MG 00), Morsoy (MG 00), and Capital (MG 00). Based on previous and current research on disease tolerance, it was concluded that commercial soybean cv. Rubin (MG II) is favorable for sustainable soybean production in the agroecological environment in southeastern Europe. This is especially important for organic and low-input production, since disease tolerance is crucial for minimizing the gap between yield potential and actual yield. Furthermore, results from this research are valuable to soybean breeders who should include tolerant cultivars in breeding programs, because tolerant cultivars secure soybean sustainable production and are the best control approach for diseases caused by *Diaporthe* species.

## Figures and Tables

**Table 1:** MDA content (nmol MDA/g FW) in leaves of soybean plants infected with *Diaporthe* isolates by three different inoculation methods (mycelium contact, toothpick, and plug).

	Mycelium Contact	Toothpick Method	Plug Method
Control	36.27 ± 0.71 ^a^	45.13 ± 1.51 ^d^	43.71 ± 0.54 ^c^
*D. aspalathi*	36.27 ± 0.71 ^a^	57.73 ± 1.13 ^h^	46.10 ± 0.95 ^e^
*D. caulivora*	54.25 ± 1.32 ^g^	51.26 ± 1.44 ^f^	58.52 ± 3.02 ^i^
*D. longicolla*	38.29 ± 2.36 ^b^	61.54 ± 0.93 ^j^	64.09 ± 1.41 ^k^

The data are mean values ± standard error; ^a–k^ Values without the same superscripts within each column differ significantly (*p* < 0.05).

**Table 2:** The activity of the antioxidant enzymes SOD, CAT, and PX (U/g FW), the GSH quantity (µmol GSH/g FW), and the MDA content (nmol MDA/g FW) in the leaves of soybean plants (cv. Sava) infected with *Diaporthe* isolates by the mycelium contact inoculation method.

	SOD	CAT	PX	GSH	LP
Control	88.10 ± 16.26 ^b^	1.42 ± 0.15 ^a^	25.48 ± 0.14 ^d^	10.15 ± 0.15 ^bc^	62.75 ± 0.52 ^c^
*D. aspalathi*	95.94 ± 20.26 ^b^	1.28 ± 0.15 ^a^	42.46 ± 0.16 ^b^	8.44 ± 0.15 ^b^	79.31 ± 0.51 ^b^
*D. caulivora*	57.51 ± 7.33 ^b^	1.16 ± 0.15 ^a^	36.79 ± 0.16 ^a^	14.52 ± 0.15 ^d^	73.01 ± 0.56 ^bc^
*D. eres*	163.39 ± 12.69 ^a^	1.34 ± 0.16 ^a^	32.11 ± 0.14 ^a^	20.20 ± 0.16 ^a^	94.21 ± 11.19 ^a^
*D. gulyae*	73.20 ± 10.77 ^b^	0.91 ± 0.17 ^a^	68.08 ± 0.17 ^c^	8.75 ± 0.17 ^bc^	73.59 ± 2.66 ^bc^
*D. longicolla*	81.83 ± 11.27 ^b^	1.12 ± 0.15 ^a^	40.18 ± 0.15 ^a^	12.82 ± 0.15 ^c^	71.77 ± 0.72 ^bc^

The data are mean values ± standard error; ^a–d^ Values without the same superscripts within each column differ significantly (*p* < 0.05).

**Table 3:** The activity of the antioxidant enzymes SOD, CAT, and PX (U/g FW), the GSH quantity (µmol GSH/g FW), and the MDA content (nmol MDA/g FW) in the leaves of soybean cultivars from different maturity groups (MG) infected with *D. eres* by the mycelium contact inoculation method.

	MG		SOD	CAT	PX	GSH	LP
Favorit	000	C	17.11 ± 4.71 ^a^	0.67 ± 0.07 ^a^	16.43 ± 0.79 ^a^	1.73 ± 0.03 ^a^	278.80 ± 6.50 ^a^
		T	26.31 ± 0.74 ^b^	0.61 ± 0.13 ^a^	17.61 ± 0.99 ^a^	1.77 ± 0.09 ^a^	268.63 ± 4.79 ^a^
Altona	00	C	22.09 ± 0.95 ^a^	1.11 ± 0.11 ^a^	18.36 ± 0.83^a^	2.10 ± 0.07 ^a^	283.89 ± 3.38 ^a^
		T	22.76 ± 1.37 ^a^	0.77 ± 0.04 ^a^	18.63 ± 0.79 ^a^	2.03 ± 0.07 ^a^	267.29 ± 7.05 ^a^
Capitol	00	C	29.42 ± 1.25 ^a^	2.42 ± 0.13 ^a^	25.56 ± 1.12 ^a^	1.79 ± 0.11 ^a^	346.41 ± 1.36 ^a^
		T	19.88 ± 0.88 ^b^	0.79 ± 0.10 ^b^	16.51 ± 0.89 ^b^	1.54 ± 0.01 ^a^	275.51 ± 1.01 ^b^
Chico	00	C	15.16 ± 2.70 ^a^	0.76 ± 0.09 ^a^	14.22 ± 1.11 ^a^	1.82 ± 0.06 ^a^	224.96 ± 6.44 ^a^
		T	21.11 ± 1.32 ^a^	1.25 ± 0.22 ^b^	13.28 ± 1.34 ^a^	1.49 ± 0.20 ^a^	211.50 ± 8.57 ^a^
McCall	00	C	32.94 ± 0.84 ^a^	2.56 ± 0.21 ^a^	31.81 ± 3.77 ^a^	2.11 ± 0.19 ^a^	231.09 ± 2.24 ^a^
		T	16.19 ± 1.89 ^b^	0.55 ± 0.10 ^b^	17.77 ± 2.02 ^b^	1.13 ± 0.09 ^b^	160.64 ± 2.47 ^b^
Morsoy	00	C	31.54 ± 1.55 ^a^	1.85 ± 0.13 ^a^	14.47 ± 0.59 ^a^	1.67 ± 0.14 ^a^	276.41 ± 4.24 ^a^
		T	21.36 ± 1.63 ^b^	0.47 ± 0.05 ^b^	9.07 ± 0.43 ^b^	1.18 ± 0.01 ^b^	232.89 ± 1.64 ^b^
Strain	00	C	17.25 ± 0.90 ^a^	0.48 ± 0.06 ^a^	11.94 ± 0.49 ^a^	1.37 ± 0.10 ^a^	174.85 ± 8.55 ^a^
		T	19.91 ± 1.12 ^a^	0.54 ± 0.06 ^a^	11.31 ± 0.60 ^a^	1.81 ± 0.14 ^a^	291.67 ± 5.10 ^b^
Atlas	0	C	27.32 ± 2.59 ^a^	1.38 ± 0.13 ^a^	22.59 ± 0.50 ^a^	1.92 ± 0.12 ^a^	264.74 ± 1.03 ^a^
		T	22.82 ± 1.09 ^a^	0.73 ± 0.10 ^b^	19.47 ± 0.45 ^a^	1.08 ± 0.05 ^b^	435.11 ± 8.72 ^b^
CX134	I	C	20.11 ± 1.80 ^a^	0.55 ± 0.02 ^a^	19.44 ± 0.77 ^a^	1.49 ± 0.08 ^a^	234.83 ± 1.50 ^a^
		T	29.03 ± 2.57 ^b^	0.81 ± 0.18 ^a^	20.93 ± 1.02 ^a^	2.03 ± 0.17 ^b^	272.37 ± 2.23 ^b^
Lakota	I	C	22.57 ± 1.33 ^a^	0.62 ± 0.08 ^a^	19.10 ± 0.79 ^a^	1.37 ± 0.21 ^a^	177.99 ± 2.98 ^a^
		T	17.09 ± 2.53 ^a^	1.12 ± 0.06 ^b^	17.82 ± 0.30 ^a^	1.73 ± 0.23 ^a^	173.95 ± 7.20 ^a^
Victoria	I	C	10.15 ± 1.30 ^a^	0.23 ± 0.04 ^a^	8.96 ± 0.34 ^a^	1.19 ± 0.20 ^a^	241.26 ± 1.22 ^a^
		T	26.73 ± 3.20 ^b^	0.71 ± 0.13 ^b^	22.69 ± 0.77 ^b^	1.65 ± 0.16 ^b^	431.82 ± 1.22 ^b^
Rubin	II	C	24.55 ± 0.50 ^a^	0.49 ± 0.08 ^a^	18.05 ± 0.46 ^a^	2.72 ± 0.24 ^a^	427.18 ± 7.18 ^a^
		T	22.90 ± 0.95 ^a^	0.60 ± 0.10 ^a^	14.47 ± 1.40 ^a^	1.57 ± 0.16 ^b^	420.45 ± 7.98 ^a^

The data are mean values ± standard error; ^a,b^ Values without the same superscripts within the control (C) and treatment (T) lines differ significantly (*p* < 0.05).

## Data Availability

Data supporting the reported results are linked with the EU Horizon 2020 Project—ECOBREED—Increasing the efficiency and competitiveness of organic crop breeding under Grant No: 771367 and can be found at the open repository Zenodo (https://zenodo.org/communities/ecobreed/?page=1&size=20 (accessed on 1 July 2023)).
